# An improved GBSO-TAENN-based EEG signal classification model for epileptic seizure detection

**DOI:** 10.1038/s41598-024-51337-8

**Published:** 2024-01-08

**Authors:** M. V. V. Prasad Kantipudi, N. S. Pradeep Kumar, Rajanikanth Aluvalu, Shitharth Selvarajan, K Kotecha

**Affiliations:** 1 Symbiosis Institute of Technology, Symbiosis International (Deemed) University, Pune, 412115 India; 2 S.E.A College of Engineering and Technology, Bengaluru, 560049 India; 3 Department of Information Technology, Chaitanya Bharathi Institute of Technology, Hyderabad, 500075 India; 4 School of Built Environment, Engineering and Computing, Leeds Beckett University, Leeds, LS1 3HE UK; 5 Department of Computer Science, Kebri Dehar University, Somali, Ethiopia; 6 Symbiosis Centre for Applied Artificial Intelligence (SCAAI), Symbiosis International (Deemed) University, Pune, 412115 India

**Keywords:** Electrical and electronic engineering, Engineering

## Abstract

Detection and classification of epileptic seizures from the EEG signals have gained significant attention in recent decades. Among other signals, EEG signals are extensively used by medical experts for diagnosing purposes. So, most of the existing research works developed automated mechanisms for designing an EEG-based epileptic seizure detection system. Machine learning techniques are highly used for reduced time consumption, high accuracy, and optimal performance. Still, it limits by the issues of high complexity in algorithm design, increased error value, and reduced detection efficacy. Thus, the proposed work intends to develop an automated epileptic seizure detection system with an improved performance rate. Here, the Finite Linear Haar wavelet-based Filtering (FLHF) technique is used to filter the input signals and the relevant set of features are extracted from the normalized output with the help of Fractal Dimension (FD) analysis. Then, the Grasshopper Bio-Inspired Swarm Optimization (GBSO) technique is employed to select the optimal features by computing the best fitness value and the Temporal Activation Expansive Neural Network (TAENN) mechanism is used for classifying the EEG signals to determine whether normal or seizure affected. Numerous intelligence algorithms, such as preprocessing, optimization, and classification, are used in the literature to identify epileptic seizures based on EEG signals. The primary issues facing the majority of optimization approaches are reduced convergence rates and higher computational complexity. Furthermore, the problems with machine learning approaches include a significant method complexity, intricate mathematical calculations, and a decreased training speed. Therefore, the goal of the proposed work is to put into practice efficient algorithms for the recognition and categorization of epileptic seizures based on EEG signals. The combined effect of the proposed FLHF, FD, GBSO, and TAENN models might dramatically improve disease detection accuracy while decreasing complexity of system along with time consumption as compared to the prior techniques. By using the proposed methodology, the overall average epileptic seizure detection performance is increased to 99.6% with f-measure of 99% and G-mean of 98.9% values.

## Introduction

Electroencephalogram (EEG) signal^1,2^ is one of the most extensively used tools for detecting human brain diseases, providing information related to brain activity's physiological states. Also, it is considered a communication medium for detecting disorders like epilepsy and brain tumor. Typically, the EEG signals^3,4^ are reasonable and non-invasive, acquiring the information for analyzing the complex behaviour of the brain. The epileptic seizure is a kind of neurological disorder^5^ that is identified based on the abnormal electrical activities of neurons. Also, it is highly essential to detect and recognize epileptic seizures to provide appropriate treatment at the time. Based on the statistical report provided by the World Health Organization (WHO), around 70 million people are affected by epileptic seizure disease^6^. So, various imaging modalities have been developed for detecting epilepsy, in which the EEG signal-based epileptic seizure detection^7^ has recently gained significant attention. Moreover, some of the automated mechanisms^8,9^ are developed in the existing works to detect epileptic seizures from EEG signals. When compared to the other approaches, machine learning algorithms are extensively used for automatic medical diagnosis systems. The typical EEG signal classification system^10^ comprises the steps of signal de-noising, feature extraction, optimization, and classification. In conventional works, different types of machine learning techniques have been employed to improve both the efficiency and accuracy of epileptic seizure detection. The de-noising is mainly performed to remove the noise contents and other artifacts present in the signal by using the filtering approaches. The feature extraction and selection techniques are used to extract the most suitable features for increasing the accuracy of the classifier. The machine learning classifiers are mainly employed to increase the effectiveness of disease detection systems. An example of a binary classification technique is the Support Vector Machine (SVM) algorithm. It is able to handle a large number of predictors despite its small sample size and high sensitivity to a very large number of variables. This is just one of its many impressive qualities. The linear decision surface is the foundation of SVM because it has the largest gap among borderline patients and can be used to isolate patient classes. Features and classes are dealt with by the Naïve Bayes (NB) algorithm. It is taking into consideration a quick algorithm that analyses all of its training datasets and needs less data for classification. NB is a probabilistic classifier that completely relies on learning while taking into account the independence of the features given the class. To categorize the samples, K-NN is regarded as a stochastic regression, non-linear, and intuitive method. Moreover, it performs well for the bigger training dataset. Weights and neurons make up the Artificial Neural Network (ANN) function. Weights transport values among neurons, whereas neurons pass input values through functions and output results.

However, the conventional optimization-based classification techniques^11–14^ limit with the following key problems:

1. Difficult to understand the system design
2. Complexity in mathematical modelling and computations.
3. Requires increased time consumption for training and testing the features.
4. Increased misprediction rate and error rate.
5. High dimensionality of features.
6. Inability to handle large dimensional datasets.
7. Overfitting and reduced convergence rate

Therefore, the proposed work intends to develop a new prediction system for accurately identifying epileptic seizure from the given EEG signals. In the proposed detection framework, simple and advanced feature extraction, optimization, and classification techniques are employed to predict the seizure with minimal computational time. The complexity of classification is minimized by optimally choosing the features according to the global best solution. In addition to that, it helps to reduce the time consumption of classification with increased prediction accuracy.

### Contributions of the proposed work

The significant contributions behind this research work are as follows,

1. An automated and accurate epileptic seizure detection system is designed to identify EEG signal abnormalities by using advanced bio-inspired optimization and deep learning classification techniques.
2. The quantitative information is obtained from the EEG signals with the help of Fractal Dimension (FD) based feature extraction methodology.
3. The optimal number of features are identified and selected based on the optimal position updation of grasshoppers using the GBSO technique. Also, the random jumping strategy is obtained from both the local and global optimal values.
4. The GBSO-TAENN improves the seizure detection scheme's overall performance by identifying original features and optimal hyper-parameters like dropout, decay, leaving and cosine similarity. These parameters are mainly considered for computing the loss function, which can be utilized for training the classifier with reduced computational complexity.
5. Moreover, the different EEG datasets, such as the University of Bonn and CHB-MIT, have been utilized to test the proposed scheme's effectiveness and accuracy during the experimental validation. These datasets comprise more complex cases of difficult signals to handle.

The other sections of this paper are structuralized as follows: section “Related works” investigates the conventional EEG signal detection and classification techniques with their benefits and demerits. Then, section “GBSO-TAENN-based seizure prediction” describes the proposed GBSO-TAENN-based seizure detection system with its detailed algorithmic and flow illustrations. The performance and comparative analysis of both existing and proposed techniques are validated in section “Results and discussion”. Finally, the overall obtainments of this paper is summarized with its future scope in Section “Conclusion”.

## Related works

This section reviews the conventional algorithms related to preprocessing, feature extraction, optimization, and classification for EEG-based epileptic seizure detection. Also, it discusses the working strategy of each technique with respect to its advantages and disadvantages.

Wang et al.^15^ introduced a new Time-Varying (TV) model with Multi-Wavelet Basis Function (MWBF) approach for detecting epileptic seizures from EEG signals. The main aim of this paper was to obtain an improved detection of a seizure by incorporating the functionalities of TV auto-regression, MWBF, and Ultra Regularized Orthogonal Forward Regression (UROFR) models. The Principle Component Analysis (PCA) based optimization model was employed to reduce the dimensionality of features based on the optimal fitness function. The significant advantages of this work were better classification performance and detection efficiency. However, it limits the issues like high complexity in algorithm design and more time consumption for processing. Alickovic et al.^16^ utilized a combination of Discrete Wavelet Transform (DWT), and wavelet-packed decomposition models for automatically detecting epileptic seizures from the given EEG signals. This system comprises the stages of signal de-noising, decomposition based on the empirical model, relevant feature extraction and classification. The empirical decomposition model was mainly utilized to decompose the signals based on the local minima and maxima values. Then, the transformation technique could be applied to construct various sub-bands of signals based on the estimated coefficients. Moreover, the performance of four different machine learning algorithms, such as SVM, k-NN, MLP, and RF, were compared based on the overall detection accuracy. This classification framework has the ability to process a large number of datasets with an improved performance rate.

Boubchir, et al.^17^ investigated the performance of different feature extraction techniques used for EEG signal processing, which comes under the categories of time-domain and frequency-domain. Then, some widely used machine learning techniques have been used to classify the signals based on the extracted features. It includes mean, variance, kurtosis, skewness, minimum, maximum, energy, peak frequency, amplitude, and mobility. Yet, this detection system is required to estimate the detection efficiency of classification with a large amount of data. Solaija, et al.^18^ suggested a new feature selection mechanism named Dynamic Mode Decomposition (DMD) for reducing the dimensionality of features used for EEG signal classification. The steps involved in this system design were preprocessing, feature extraction, selection, classification, and post-processing. The channel selection was performed during the signal processing, and the DMD and curve length features were extracted from the de-noised signal. After that, the RUSBoost decision tree technique was employed to train the classifier based on the feature values. Finally, the signal post-processing has been performed to decide whether the input signal is normal or seizure-detected based on the consequences of several epochs. It offered the benefits of reduced complexity and time consumption for processing the signals. Still, it limits the issues of reduced accuracy and detection efficiency in classification.

Jaiswal and Banka^19^ developed two different feature extraction approaches, such as Sub pattern-based PCA (SpPCA) and Cross-Sub pattern correlation-based PCA (SubXPCA) to improve the efficiency of automatic epileptic seizures detection. Also, the SVM classification technique was employed to classify the seizure-affected signal based on the set of extracted feature vectors. The merits of these techniques were reduced space and time complexities during feature extraction. Wang et al.^20^ implemented a new detection methodology based on multiple feature extraction and classification to predict an epileptic seizure. Here, the Daubechies wavelet threshold technique was employed to normalize the input signal; then, the wavelet decomposition has been performed to obtain the sub-bands of the de-noised signal. After that, the PCA-based optimization technique was utilized to reduce the dimensionality of features by selecting the best fitness function. Finally, the machine learning classifier was used to categorize whether the signal is normal or abnormal based on the selected feature values. Kalbkhani and Shayesteh^21^ implemented a kernel Principal Component Analysis (PCA) method for reducing the dimensionality of feature vectors used in the EEG signal epileptic seizure detection system. Here, the Stockwell transformation was performed to partition the frequency bands, then the feature vectors were calculated with respect to the amplitude distribution. Based on these features, the most optimal feature values were selected using kPCA, and the k-nearest neighbor classification technique was employed to categorize the signals as Healthy, Interictal, and Ictal. Moreover, it obtained the advantages of reduced time complexity and increased classification accuracy.

Atal et al.^22^ developed an automatic classification system for discovering the abnormalities of the input EEG signals. Here, an Enhanced Curvelet Transform (ECT) technique was applied to denoise the signal by excluding the irrelevant positions and noisy contents. Sequentially, the quantitative information from the preprocessed signal was extracted by applying the graph and texture-based feature extraction mechanism. Then, the Grey Level Co-occurrence Matrix (GLCM) technique was deployed to extract the statistical information for simplifying the process of abnormality detection. Finally, the Random Forest (RF) classification technique uses the selected feature vectors to label the signal as normal or abnormal. Tsiouris et al.^23^ designed an automatic epileptic seizure recognition system using the unsupervised machine learning approach, where the signals with epileptic abnormality were detected and isolated with increased accuracy. The authors validated the seizure detection performance of the suggested unsupervised learning methodology using different measures like time, false detection rate, sensitivity, specificity, and etc.

Chen et al.^24^ applied the nearest neighbours classification with the Fast Fourier Transform (FFT) technique to detect epileptic seizures from the EEG signals. The main intention of this paper was to obtain an improved classification performance with reduced detection time. In paper^25^, a complete ensemble empirical model decomposition model for classifying the given EEG signal as seizure or normal. It incorporates the processes of segmentation, signal decomposition, feature extraction, training/testing, and signal classification. Also, this work employed an adaptive boost classification technique to improve the accuracy and efficiency of the seizure detection system.

Zhou et al.^26^ suggested a Convolutional Neural Network (CNN) technique for recognizing epileptic seizures from the EEG signals, where the signal was classified into interictal, preictal, and ictal. For this analysis, the CHB-MIT database was utilized to test the average accuracy of this model with respect to these three classes. Still, this work has the major drawbacks of increased complexity in design and high time consumption for processing. Dash et al.^27^ utilized a Hidden Markov Model (HMM) classification technique to detect the input EEG signal's seizure and non-seizure activities. This system comprises the working stages of preprocessing, decomposition, spectral density analysis, clustering and classification. The k-means clustering technique helps obtain an improved classification performance by identifying the similar data points with respect to the distance vector^28–30^. However, this detection system lacks the issues of reduced robustness and high noisy contents, which affects the entire classification performance.

From the survey^31–37^, it is studied that the existing works utilized different types of machine-learning classification techniques for detecting the epileptic seizure from the input signals with respect to varying classes. Yet, some of the limitations could degrade the effectiveness and accuracy of the seizure detection system, which include:

- High computational complexity.
- Reduced detection efficiency and accuracy.
- Very sensitive to artifacts.
- High time cost.

Thus, this research expects to design an effective signal classification framework for an accurate prediction and classification of epileptic seizures.

## GBSO-TAENN-based seizure prediction

This sector delivers the complete depiction of the proposed GBSO-TAENN classification system with its algorithmic and flow illustrations. The main motive of this paper is to precisely detect the EEG signal as to whether normal or seizure-affected by using advanced optimization and classification techniques. The motivation of this research work is to develop a new and computationally effective disease prediction framework for epileptic seizure detection and classification. For improving better performance, advanced optimization and classification methodologies are implemented in the proposed framework. In order to attain better accuracy and performance, the Finite Linear Haar wavelet-based Filtering (FLHF) approach is used for signal preprocessing and normalization. Then, the Fractional Dimension (FD) based feature extraction is performed to obtain the suitable features from the preprocessed signal. Consequently, the GBSO algorithm is applied to reduce feature dimensionality, where the unique inertia weight updation is performed to improve the performance of optimization. Moreover, the deep learning model, named as, TAENN is utilized to predict the signal abnormalities with improved accuracy and reduced time consumption. The literature uses a variety of intelligence algorithms, including preprocessing, optimization, and classification, to identify epileptic seizures based on EEG readings. The main problems that most optimization techniques face are increasing computing complexity and lower convergence rates. Moreover, the challenges associated with machine learning techniques comprise a high degree of method complexity, sophisticated mathematical computations, and reduced training speed. Thus, putting into practice effective algorithms for the identification and classification of epileptic seizures based on EEG signals is the aim of the proposed work. When compared to earlier methods, the combined impact of the suggested FLHF, FD, GBSO, and TAENN models may significantly increase illness detection accuracy while lowering system complexity and time consumption.

The working stages involved in the proposed system are as follows:

- Signal de-noising and decomposition using FLHF
- FD analysis-based feature extraction
- Optimization using GBSO
- Abnormality identification based on TAENN

**Figure 1:**
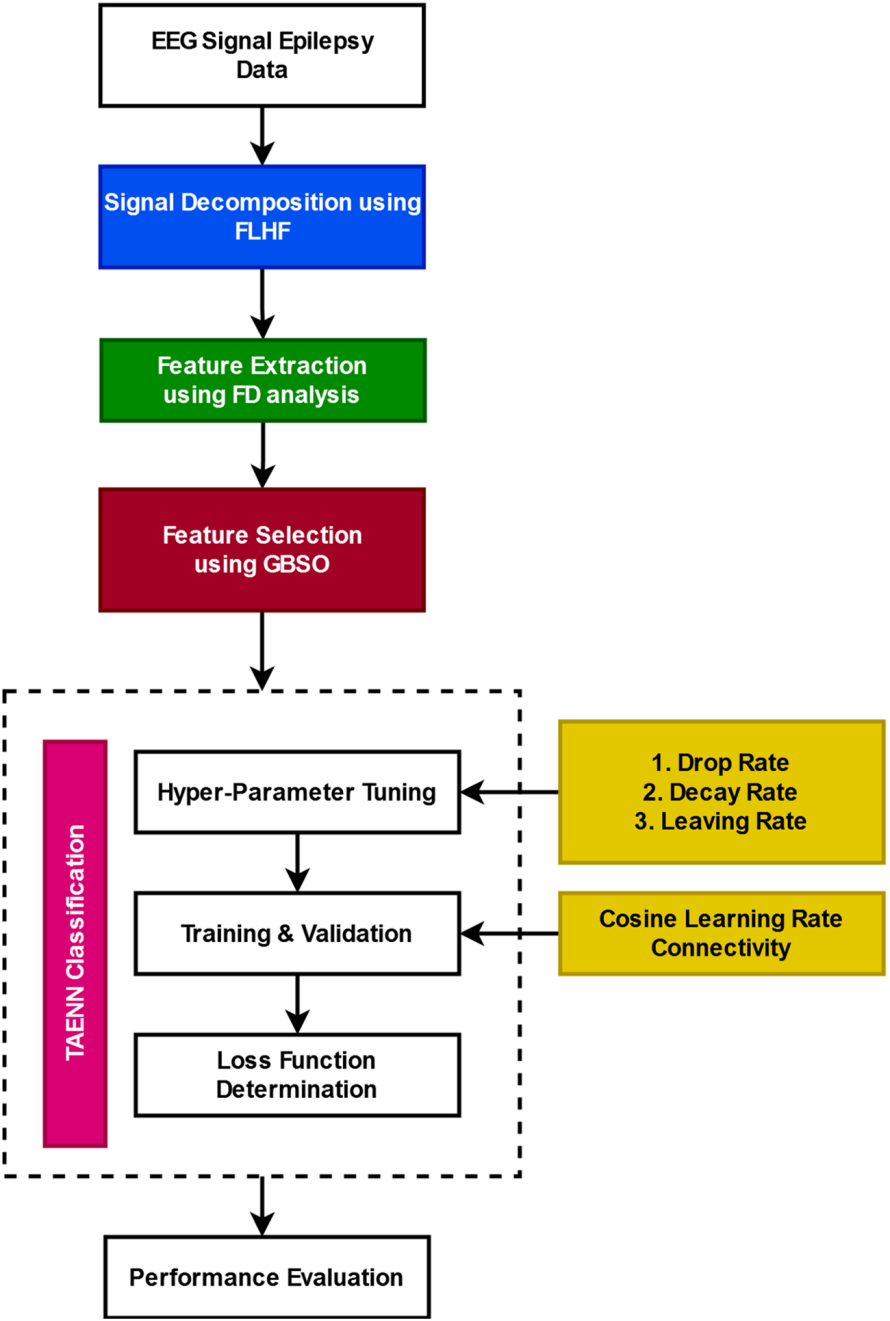
The flow of GBSO-TAENN-based epileptic seizure detection system.

In the work flow mentioned in Fig. 1, two different datasets, such as University of Bonn EEG and CHB-MIT EEG, have been used to test the proposed classification system. At first, the input EEG signal obtained from these datasets is preprocessed by eliminating the artifacts and noisy contents with the help of the FLHF technique. Then, the different types of FD features such as higuchi FD, katz FD, sevcik’s FD, instantaneous energy, teager energy and Petrosian FD are extracted from the filtered signal.

Consequently, the GBSO-based optimization technique is employed to reduce the dimensionality of features by computing the optimal fitness function. Based on the selected feature vectors, the TAENN technique classifies the given signal as to whether normal or seizure affected. The hyperparameter tuning is performed with the dropout, decay, and learning rate measures. The major advantages of the proposed GBSO-TAENN technique are high classification accuracy, reduced computational complexity, increased efficacy, reduced overfitting, and better prediction performance. Moreover, an advanced deep learning mechanism is utilized for classification, where the hyper-parameters like drop, decay, leaving, and cosine learning are computed for computing the loss function. Then, this value can be utilized for training the classifier, and it also helps reduce the classification system's complexity.

### Preprocessing

The EEG signal preprocessing is one of the essential stage in medical diagnosing systems, where the noise/artifacts removal is performed for de-noising the signal. In this stage, the signal decomposition is mainly performed to reduce the complexity of input EEG signals, which also helps to improve both the detection and classification performance rate. Due to the non-linearity nature of EEG signals, signal decomposition is used for accurately detecting epileptic seizures. For this purpose, the Finite Linear Haar wavelet-based Filtering (FLHF) approach is deployed in this work, which performs the processes of signal decomposition, artifacts/noise removal, and normalization. Typically, the haar wavelets is a type of wavelet transformation model, which converts the discrete signal into two sub-signals. Also, it is computationally fast, memory effective, and it can be easily reversible with minimal data loss. Moreover, the haar functions are treated as the orthogonal functions, which helps to estimate the frequency component of the given signal. Therefore, the proposed work intends to utilize a FLHF based filtering technique for signal preprocessing and normalization operations.

In this filtering technique, the mother wavelet transformation has been performed, where the rectangular format of mother wavelets are considered for obtaining the signal error rates. Based on that, the noise location has been identified and removed by computing the probability value. The main reason for deploying this filtering technique is to accurately detect and eliminate the noisy contents that exist in the signal by using the rectangular mother wavelets. The significant benefits of this preprocessing technique are as follows: fast computation, simple design structure, and less memory consumption by following the simple steps for noise removal.

Here, the EEG signal obtained from the given dataset is taken as the input $s_{i} \left( t \right)$ and the artifacts removed signal is produced as the output $A_{R} \left( t \right)$. Here, the mother wavelet can be defined based on the Haar wavelet function as shown below:

1 $$\tau_{{\left( {r,c,d} \right)}} \left( t \right) = \tau_{\left( c \right)} \left( {2^{r} t - n} \right)$$

where, $2^{r}$ indicates the support size of the Nyquist frequency and $\left( {r,c,d} \right)$ are the indices, $r$ is the scale index, $c$ defines the frequency shift phase parameter, and $d$ represents the time-related parameters. After that, the rectangular form is constructed from the mother wavelet as shown below:

2 $$\tau^{h} \left( t \right) = \left\{ {\begin{array}{*{20}c} {1; \;\; if 0 \le t \le 1/2} \\ { - 1; \;\; if 1/2 \le t \le 1} \\ {0; \;\; for\; all\; other t} \\ \end{array} } \right.$$

Consequently, the wavelet packet coefficients are estimated based on the coherent part $e\left( t \right)$ of sequence $s_{i} \left( t \right)$, which is illustrated as follows:

3 $$e\left( t \right) = \mathop \sum \limits_{{r^{*} ,c^{*} }} \mathop \sum \limits_{p = 0}^{{2^{D - r} }} pc_{{(r^{*} ,c^{*} ,p)}} { }\tau_{{\left( {r,c,d} \right)}} \left( t \right)$$

where $D$ indicates the length of wavelet transformed signal described by $\log_{2} \left( n \right)$, and $n$ is the length of the sequence. Then, the measured signal $s_{i} \left( t \right)$ and noise component $e\left( t \right)$ can be differentiated for obtaining the observed sequence $A_{R} \left( t \right)$, which is illustrated as follows:

4 $$A_{R} \left( t \right) = s_{i} \left( t \right) - e\left( t \right)$$

Finally, the wavelets are rearranged with respect to the localization of the noise component, and the detected subspaces of probability is estimated as follows:

5 $$pc_{{(r^{*} ,c^{*} ,p)}} = \left| {\mathop \sum \limits_{p = 0}^{{2^{D - r} }} \left\{ {pc_{{(r^{*} ,c^{*} ,p)}} } \right\}} \right|$$

The algorithmic illustration of the FLHF technique is shown below:

**Figure:**
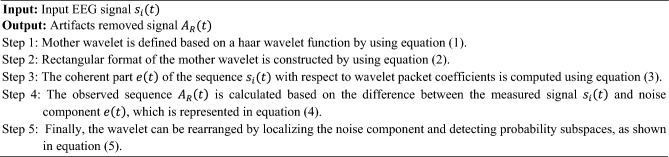
Algorithm I—Finite Linear Haar wavelet-based Filtering (FLHF)

### Feature extraction

After normalization, the features are extracted from the preprocessed signal using the Fractal Dimension (FD) analysis model^38^. The FD is a type of biological waveform that is typically employed to evaluate signal complexity because of its two main characteristics: self-similarity and irregularity. Furthermore, measuring FD for the seizure detection system yields more effective results than the currently used feature extraction methods. At this point, the normalized signal is used to extract the various FD properties, such as teager energy, instantaneous energy, katz, sevcik's, and higuchi. The Higuchi FD is mostly based on discrete time series signals and is used to evaluate the waveform's fractal dimension. The Kalz-FD is therefore better suited to handling problems involving the unit-making technique, where a variable is employed to verify the average distance between the successive points. Furthermore, the hausdorff dimension is used to estimate the approximate value of Sevcik’s FD. As such, the log-based energy, or teager, is calculated based on the signal's amplitude and frequency fluctuations.

Here, the Higuchi FD feature is considered for accurately estimating the signals, katz FD is mostly concentrated for obtaining the best aggregation degree, and the sevrick’s energy is computed for analyzing the variations in the energy distribution of each peak of the signal. Similarly, the teager energy is used to estimate the IMF component of signal for analyzing the extreme across two successive zero crossings, and the Petrosian feature has been used to analyze the exceeds of the standard deviation magnitude of each signal peak.

Initially, the FD waveform of the given input signal can be defined as follows:

6 $$FD_{T} = \frac{{\log_{10} \omega }}{{\log_{10} d_{1n} }}$$

where $\omega$ indicates the wavelength and, $d_{1n}$ is the Euclidean distance between wave points. Then, $FD_{T}$ is updated as follows:

7 $$FD_{T} = \max \left( {d_{1n}^{tc} \left( {1,j} \right)} \right)$$

where, $d_{1n}^{tc}$ represents the distance value, and $j$ indicates the maximum range obtained from the starting point. Consequently, the Higuchi FD can be extracted as follows:

8 $${\text{X}}_{{\text{j}}}^{{\text{n}}} = {\text{y}}\left( {\text{n}} \right),{\text{y}}\left( {{\text{n}} + {\text{j}}} \right),{\text{y}}\left( {{\text{n}} + 2{\text{j}}} \right),{\text{y}}\left( {{\text{n}} + 3{\text{j}}} \right), \ldots ,{\text{y}}\left( {{\text{n}} + \left[ {\frac{{{\text{D}} - {\text{n}}}}{{\text{j}}}} \right]{\text{j}}} \right)$$

where, $n = 1,2,3, \ldots ,j$ and $\left[ {\frac{D - n}{j}} \right]$ is the Gauss notation, *j* and* n* indicated as the integers. Then, the value of *j* can be used to find out the starting and interval time values, and the length of $X_{j}^{n}$ can be updated at every time as shown in below:

9 $$A\left( j \right) = \frac{1}{j}\left[ {\mathop \sum \limits_{t = 1}^{{\left[ {\frac{D - n}{j}} \right]}} \left| {X\left( {n + tj} \right) - X\left( {n + \left( {t - 1} \right)j} \right)} \right|\frac{D - 1}{{\left[ {\frac{D - n}{j}} \right]j}}/j} \right]$$

where, $\frac{D - 1}{{\left[ {\frac{D - n}{j}} \right]j}}$ indicates the normalization factor with respect to the length of FD. The average value of $A\left( j \right)$ over *j* sets (i.e.) $A_{n} \left( j \right)$ is updated as follows:

10 $$A\left( j \right) = \mathop \sum \limits_{n = 1}^{j} A_{n} \left( j \right){ }$$

Subsequently, the Katz FD can be extracted with respect to the mean distance $d_{avg}$ of successive points as shown in below:

11 $$FD_{T} = \frac{{\log_{10} \left( {\frac{\omega }{{d_{avg} }}} \right)}}{{\log_{10} \left( {\frac{{d_{1n} }}{{d_{avg} }}} \right)}} = \frac{{\log_{10} h}}{{\log_{10} \left( {\frac{{d_{1n} }}{\omega }} \right) + \log_{10} h}}$$

where the value of $h$ can be divided the states of $\omega$ with respect to the number of successive points. Then, the Sevcik’s FD is extracted based on the Hausdorff distance $H_{d}$, which is represented as follows:

12 $$H_{d} = \mathop {\lim }\limits_{\gamma \to 0} \frac{{ - {\text{log }}\left( {D\left( \gamma \right)} \right)}}{{{\text{log }}\left( \gamma \right)}}$$

where $D\left( \gamma \right)$ indicates the total radius ε required for FD, then,

the waveform with the length *L* can be defined as follows:

13 $$FD_{T} = \mathop {{\text{lim}}}\limits_{\gamma \to 0} - \frac{{{\text{log }}\left( L \right) - {\text{log }}\left( {D\left( \gamma \right)} \right)}}{{{\text{log }}\left( \gamma \right)}}$$

where, $D\left( \gamma \right) = L/2\gamma$. Based on signal normalization, the unit vector can be visualized in $D \times D$ cells, which is shown in below:

14 $$FD_{T}^{s} = \mathop {{\text{lim}}}\limits_{{D^{\prime} \to \infty }} \left[ {1 + \frac{{{\text{log }}\left( L \right) - {\text{log }}\left( 2 \right)}}{{{\text{log }}\left( {2\left( {D - 1} \right)} \right) }} } \right]$$

where, $FD_{T}^{s}$ equals to the fractal dimension $FD_{T}$ and its approximation is boosted as $D \to \infty$. In addition to that, the signal instantaneous energy can be defined based on the distribution on each band, which is illustrated as follows:

15 $$E_{I} = \log_{10} \left( {\frac{1}{{N_{i} }}\mathop \sum \limits_{t = 1}^{{N_{i} }} (w_{i} \left( t \right)^{2} } \right)$$

where, $N_{i}$ represents the amount of signal samples, and $w_{i}$ indicates the weight value of vector at each sample. Subsequently, the teaser energy can be represented based on the values of amplitude and frequency of signal as shown in below:

16 $$E_{I} = \log_{10} \left( {\frac{1}{{N_{i} }}\mathop \sum \limits_{t = 1}^{{N_{i} - 1}} |w_{i} \left( t \right)^{2} - w_{i} \left( {t - 1} \right)*w_{i} \left( {t + 1} \right)| } \right)$$

Finally, the Petrosian FD is computed as follows:

17 $$FD_{petr} = \frac{{log_{10} m}}{{log_{10} m + log_{10} \left( {\frac{m}{{m + 0.4N_{i} }}} \right)}}$$

The above-extracted features help to increase the overall efficiency of signal classification. Then, the dimensionality of these features can be minimized by applying the optimization technique.

### Feature selection

To increase classification accuracy and efficiency, the best features are chosen using the GBSO technique once the set of features has been extracted. The hybrid BF and GA method known as genetically bacterial swarm optimisation (GBSO) in the current work uses three genetic operators—crossover, mutation, and selection—as well as BF's chemotactic mechanism to perform local search. Furthermore, when a person is removed from the initialization process in order to generate a new one, the course does not alter; rather, a new person is created by altering every dimension. The two stages of the GBSO strategy are as follows: the first stage concentrates on genetic selection through the use of the breeder genetic algorithm (BGA) mutation, crossover through extended initial mating, and stochastic universal sampling (SUS) method. But, it has the following problems:

1. Slow convergence
2. Due to the fixed step size, it is more complex to balance the exploration and exploitation capabilities
3. Local optimum
4. Complex design

However, the Geneticall Bacterial Swarm Optimization (GBSO) method now in use is not the same as the suggested Grasshopper Bio-Inspired Swarm Optimization (GBSO).For feature selection in the suggested work, we employed the Grasshopper Bio-Inspired Swarm Optimization (GBSO) algorithm. This method balances exploration and exploitation by updating the inertia weight based on the parameter G. Additionally, by changing one of the parameters G in the suggested GBSO model, the consistency of the search process is guaranteed. Furthermore, the suggested GBSO replicates the swarming behaviors of grasshoppers. Additionally, the grasshopper optimization method will be used in the proposed work to choose the best characteristics from the EEG input. It is a type of bio-inspired meta-heuristic optimization method that emulates the swarming behavior of grasshoppers^39^. It offers the best results and most appropriate optimal solutions when compared to other optimization strategies for resolving the provided issues. The GBSO^40^ technique is applied in this work because of its quality of solution and exploration capabilities. It effectively solves the complex optimization problem by mimicking the behavior of grasshoppers in nature. The social interaction probability is estimated in the proposed work with the social force, which is a key distinction between the existing and proposed grasshopper optimization techniques. It facilitates the achievement of the global optimal value and a higher convergence rate. In addition, it offers excellent accuracy, low exploration and increased exploitation, and ease of deployment.

Furthermore, in comparison to other optimization strategies, the searching efficiency and convergence speed are significantly higher. Based on the parameters of unity vector, social interaction, and gravitational force, the GBSO objective function is calculated. Based on the optimized value, the ideal amount of features for this model is chosen from a list of features that are available. Next, based on the ideal values of the variables employed in the optimization, the optimal cost is calculated. In this optimization technique, the input populations of the grasshoppers are updated for the prediction of best optimum value. Since the updating populations can be repeated in this case, the location can always be altered. Thus, in order to complete the feature selection process, it is helpful to obtain both the local and global optimal values. Just the local optimal value has been determined in relation to the weight function in the traditional grasshopper model. However, in the suggested model, the random jumping process has been used to compute both the local and global values. This optimization method has the following advantages: faster convergence, fewer parameters needed, manipulative searching pattern, high coverage, precise setup, and higher-quality random populations.

In this technique, the population size *N* and maximum number of iterations $I_{mx}$ are taken as the input, and the optimized value $O_{V}$ is produced as the output. After getting the input parameters, the initial population is generated as follows:

18 $$X_{j} = S_{j} + Gr_{j} + Ad_{j}$$

where, $S_{j}$ indicates the social interaction of the $j{\text{th}}$ grasshopper, $Gr_{j}$ defines the gravity force of the $j^{th}$ grasshopper, and $Ad_{j}$ represents the wind advection of the $j{\text{th}}$ grasshopper. Then, $Gr_{j}$ is defined as follows:

19 $$Gr_{j} = - g_{c} \widehat{{e_{c} }}Ad_{j} = d_{c} \widehat{{e_{w} }}$$

where, $g_{c}$ is the gravitational constant, $d_{c}$ indicates the constant drift, $e_{c}$ defines the unity vector towards the center of the earth, and $e_{w}$ Indicates the unity vector towards the direction of the wind.

As shown in Eq. (18), three key elements make up simulation of gravitational force, wind advection, and social interaction. These three elements mimic grasshopper movement. To tackle optimization problems, GBSO merely mimics the social interaction as represented in Eqs. (20) and (21). Then, the current iteration is initialized as I = 1, and the social interaction is estimated for the jth grasshopper, which is shown below:

20 $$S_{j} = \mathop \sum \limits_{j = 1, k \ne j}^{N} s_{f} \left[ {d_{jk} } \right]\widehat{{d_{jk} }}$$

21 $$s_{f} \left( r \right) = \sqrt {\frac{f}{{exp^{{\left( {{\raise0.7ex\hbox{$r$} \!\mathord{\left/ {\vphantom {r g}}\right.\kern-0pt} \!\lower0.7ex\hbox{$g$}}} \right)}} }}}$$

where, $S_{j}$ indicates the social interaction, *N* is the total number of grasshoppers in the population, $s_{f} \left( r \right)$ is the social force, $f$ is the attraction intensity, $g$ is the length of attraction scale, and $d_{jk}$ represents the distance value between $j{\text{th}}$ and $k{\text{th}}$ grasshoppers. According to where a grasshopper is in relation to nearby grasshoppers, it may experience the three forces of attraction, repulsion, and neutrality in a grasshopper swarming. Thus, the area is split into three sections: the region before the comfort zone, the area inside the comfort zone, and the area after the comfort zone. These three parameters and their forces are simulated using the aforementioned mathematical function. The function s is used to demonstrate how it affects grasshoppers' social interactions (attraction and repulsion). Here, the repulsion occurs between 0 and 2.079. When addressing optimization issues, this value can be normalized to any desired range. When the distance is 2.079, the grasshopper will be in an unforced neutral position. The attractive force increases as the grasshopper moves farther, up until around 5, when the vast distance causes the forces to decrease. In GOA, it is considered that the function s's operating range is 0–3.3.

**Figure 2:**
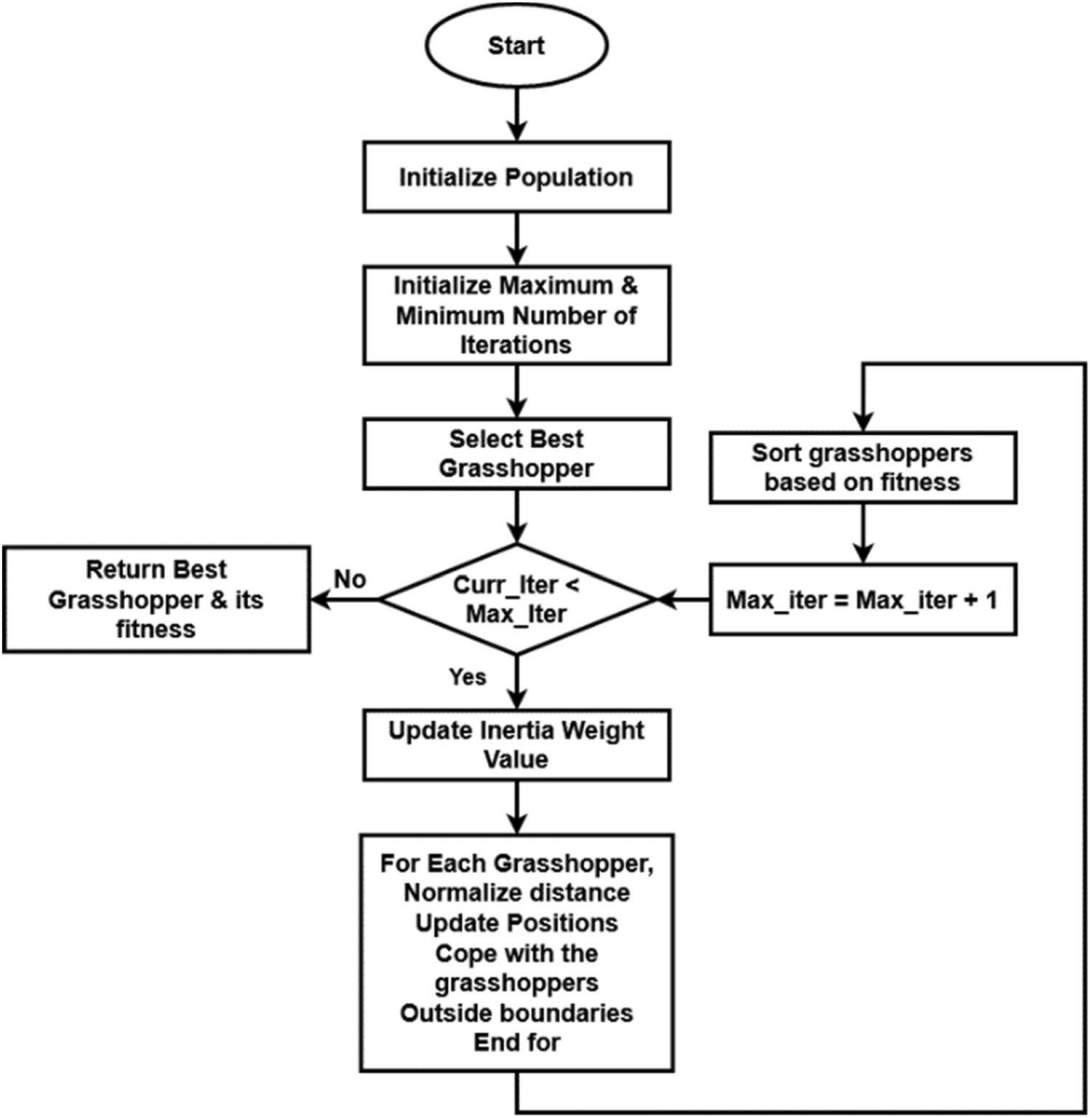
Workflow of GBSO algorithm.

Consequently, estimating the fitness function of all feature vectors is the best optimal solution. Then, the value of optimization function *G* is estimated with respect to the values of upper and lower bounds of dimension, which is shown in below,

22 $$G = G_{maxi} - it\frac{{G_{maxi} - G_{mini} }}{{it_{maxi} }}$$

where, $G$ is the optimization function, $it$ indicates the current iteration, $it_{maxi}$ is the maximum number of iterations, $G_{maxi}$ and $G_{mini}$ are maximum and minimum values of coefficients for reducing exploration, and increasing exploitation. The classic GOA algorithm relies on the parameter G to balance both exploration and exploitation, which linearly declined as iterations increased. Yet, without taking into account each grasshopper's fitness, all the grasshoppers use the same parameter. In the proposed work, a unique inertia weight calculation is performed that adopts several tactics based on the fitness value of grasshoppers. Moreover, the consistency of the searching procedure is ensured in the proposed GBSO model by replacing one of the parameter G. The work flow model of the proposed GBSO algorithm is shown in Fig. 2. Based on the following model, the inertia weight value updation is performed:

23 $$\left\{ {\begin{array}{*{20}c} {G_{i}^{k} = G_{maxi} - \left( {G_{maxi} - G_{mini} } \right) \times \left( {\frac{it}{{it_{maxi} }}} \right) \;\;if \;\;fitness\left( i \right) \ge avg} \\ {G_{i}^{k} = G_{maxi} - \left( {G_{maxi} - G_{mini} } \right) \times \left[ {\frac{2it}{{it_{maxi} }} - \left( {\frac{it}{{it_{maxi} }}} \right)^{2} } \right]\;\;if\;\; fitness\left( i \right) < avg} \\ \end{array} } \right.$$

Then, the distance values are normalized between the solutions in set X and the value of $x_{j} \in X$ is updated as follows:

24 $$x_{j} = G\left[ {\mathop \sum \limits_{j = 1, k \ne j}^{N} G\frac{{u_{b} - l_{b} }}{2}s_{f} \left[ {x_{j} - x_{k} } \right]\frac{{x_{j} - x_{k} }}{{d_{jk} }}} \right]$$

where, $x_{j}$ is the jth grasshopper, $x_{k}$ is the kth grasshopper, $u_{b}$ is the upper bound, and $l_{b}$ is the lower bound. Finally, the optimized value is obtained as $O_{V} = x_{j}$, which can be used for improving the performance of classification. Before applying an optimization technique, the total number of features is 724, and after applying the proposed GBSO technique, the number of features are reduced to 497.

**Figure:**
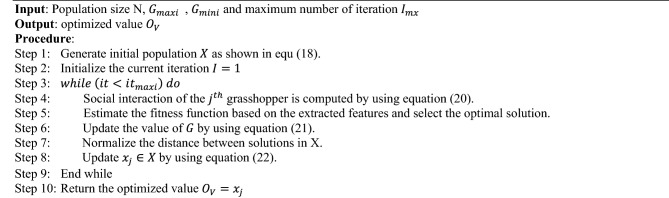
Algorithm II—Grasshopper Bio-Inspired Swarm Optimization (GBSO)

### Classification

After selecting the optimal features, the TAENN technique is implemented to detect the epileptic seizure from the input signal sequence accurately. This technique is developed based on the standard Deep Neural Network (DNN), Spatio-Temporal Neural Network (i.e. CNN and gated recurrent unit),^13,41,42^ and back propagation algorithm, which offers a large set of functions for analyzing the patterns of seizure-affected signals. The proposed TAENN is able to effectively deal with the temporal correlation that exists within the input time series because of the directional circulation mechanism. By applying this method, the temporal information of EEG sequences can be processed effectively on the foundation of extracting EEG spatial information. This layered architecture comprises the three layers of input state, hidden state and output state. Typically, selecting the optimal values for tuning the hyper-parameters is one of the most difficult and challenging tasks in the deep learning model, because it can directly degrades the learning model's performance, and are more significant to the datasets. In this classification algorithm, the hyper-parameters such as dropout, decay and learning rate are tuned for proper training and testing.

At first, the dropout parameter is used to eliminate the randomly chosen neurons for training the learning model with reduced data specialization. Also, it mainly intends to increase the generalization ability of the classification network by utilizing the more dependable neurons for training. The incorporation of dropout technique in the input layer can increase the loss of informative attributes, but the use of dropout technique in the hidden layer ensures increased classification effectiveness. Hence, it is more essential to deploy the dropout rates based on the clear analysis of the specific model and level of learning. Generally, the training data overfitting can happen due to the large weight values of the network with increased variability and complexity. In order to solve this issue, the L1 regularization has been used, which increases the generalization capability of network training data by making the smaller weight values with zero mean distribution. The main advantage of using this model is, it effectively improves the robustness by optimally selecting values.

Moreover, the cosine learning rate is used to obtain the minimal loss value by discovering the local minimums, which incorporates the parameters of decay and learning rate. The selection of these parameters highly depends on their topological structure, and it is more important to select the most suitable values for these parameters. Because the better parameter identification with the best local minimum helps to reduce the computational complexity with high generalization ability. Hence, these hyper parameters have been utilized in this classification system to enhance the training data model’s transient behaviour.

25 $$X = \left\{ {x_{1} ,x_{2} , \ldots ,x_{n} } \right\}$$

where, $X$ indicates the entire feature set of EEG signal, $x_{n}$ represents the number of EEG signal (i.e. the extracted feature values of nth EEG signal). Then, the hidden state of the network is defined as follows:

26 $$h_{n} = \left\{ {\begin{array}{*{20}l} {0~} \hfill & {~if~\;n = 0} \hfill \\ {\tau \left( {h_{{n - 1}} ,x_{n} } \right)~} \hfill & {~else} \hfill \\ \end{array} } \right.$$

where, $\tau$ represents the nonlinear function such as the hyperbolic tangent function, and the rule for the hidden state is updated as follows:

27 $$h_{n} = \tau \left( {C_{i} x_{n} + U_{p} h_{n - 1} } \right)$$

where, $C_{i}$ indicates the coefficient matrix of input and $U_{p}$ is the coefficient matrix of present hidden state. In this mechanism, the essential hyper-parameters such as dropout rate, decay rate, and learning rate. In which, the dropout rate is mainly used to eliminate the random neurons during the training process, and helps to analyze the network with less specialization. The decay is used to stabilize the network with reduced weight value, and to reduce the overfitting of training data with improved generalization capability. Then, the learning rate is utilized for discovering the local minimum as shown in below:

28 $$\delta = e^{{ - \theta_{n} Ep}} (\rho_{n} + \frac{1}{2}\left( {\vartheta_{n} - \rho_{n} } \right)\left[ {1 + \cos \left( {\frac{Ep}{{Ep_{i} }}\pi } \right)} \right]$$

where, $\theta_{n}$ defines the exponential decay rate, $\rho_{n}$ is the minimum learning rate, $\vartheta_{n}$ indicates the maximum learning rate, $Ep_{i}$ is the total number of epochs, and $Ep$ defines the current epochs. Consequently, the conditional probability distribution is estimated as follows:

29 $$p\left\{ {x_{1} ,x_{2} , \ldots ,x_{n} } \right\} = \tau \left( {h_{n} } \right)$$

Then, the activation function is estimated for the network by using,

30 $$h_{n} = o_{n} {\text{tanh}}\left( {c_{n} } \right)$$

where, ${\text{tanh}}\left( . \right)$ indicates the hyperbolic tangent function, and $o_{n}$ is the output layer of the network. After that, the output layer of the network can be updated based on the weight matrix, which is illustrated as follows:

31 $$o_{n} = \delta \left( {W^{oi} x_{n} + W^{ho} h_{n - 1} + W^{om} m_{n} } \right)$$

where, $\delta \left( . \right)$ is the logistic sigmoid function, $W^{oi}$ represents the weight matrix of input and output state, $W^{ho}$ defines the weight matrix for hidden and output state, and $W^{om}$ represents the weight matrix for memory and output state. The memory state $m_{n}$ is computed as follows:

32 $$m_{n} = tanh\left( {W^{mi} x_{n} + W^{mh} h_{n - 1} } \right)$$

where, $W^{mi}$ defines the weight matrix of input and output state, and $W^{mh}$ indicates the weight matrix of hidden and output state. Then, the activation function is reformulated based on the learnable parameters, which are shown below:

33 $$f\left( {h_{n} } \right) = \left\{ {\begin{array}{*{20}l} {\tanh \left( {h_{n} } \right)} \hfill & {if\;~h_{n} > 0} \hfill \\ {\gamma _{n} \tanh \left( {h_{n} } \right)~} \hfill & {~if\;~h_{n} \le 0} \hfill \\ \end{array} } \right.$$

where, $\gamma_{n}$ is the learnable parameter. Simultaneously, the parameters are optimized in the network based on the chain rules formed by $\gamma_{n}$, which is defined as follows:

34 $$\frac{\partial L}{{\partial \gamma_{n} }} = \mathop \sum \limits_{{h_{n} }} \frac{\partial L}{{\partial f\left( {\gamma_{n} } \right)}} \frac{{\partial f\left( {\gamma_{n} } \right)}}{{\partial \gamma_{n} }}$$

where, $\frac{\partial L}{{\partial f\left( {\gamma_{n} } \right)}}$ is the gradient back propagated from the deeper layer of the activation function as shown in below:

35 $$\frac{{\partial f\left( {\gamma _{n} } \right)}}{{\partial \gamma _{n} }} = \left\{ {\begin{array}{*{20}l} 0 \hfill & {~if\;~h_{n} > 0} \hfill \\ {\tanh \left( {h_{n} } \right)~} \hfill & {~if\;~h_{n} \le 0} \hfill \\ \end{array} } \right.$$

Finally, the predicted label *y* can be obtained by using,

36 $$y = W^{ho} h^{K} + b_{o}$$

where, $W^{ho}$ is the output-hidden weight matrix, $h^{K}$ indicates the hidden vector sequence, and $b_{o}$ defines the bias vector of the output layer. The algorithmic steps involved in the proposed TAENN technique is illustrated below:

**Figure:**
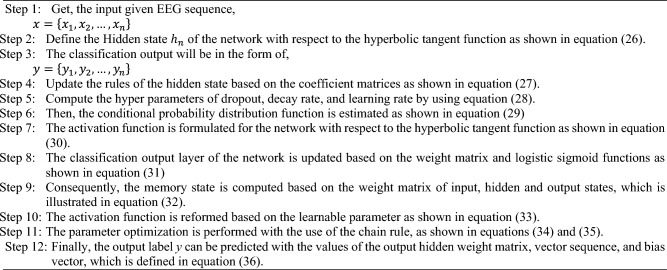
Algorithm III—Temporal Activation Expansive Neural Network (TAENN)

## Results and discussion

This section presents the performance analysis of both existing and proposed epileptic seizure detection techniques with respect to varying performance measures. The major contribution of this work is to accurately detect the epileptic seizure from the input EEG signals by using an advanced optimization and classification methods. Also, it motivates to increase the detection accuracy with minimal computational complexity. For this purpose, a combination of GBSO-TAENN mechanisms are employed in the proposed work, which predicts the input signal as whether normal or seizure affected.

### Dataset details

Two distinct benchmark datasets, namely Bonn University^43^ and CHB-MIT^44^, are used in this evaluation, in which the Children’s Hospital Boston’s CHB-MIT database was the one used in this investigation.

#### CHB-MIT

EEG recordings from 24 pediatric patients who were having uncontrollable seizures are included in the collection. This collection contains 916 h of EEG data and 23 samples of EEG recordings from 22 people, ages ranging from 1.5 to 22 years. A continuous EEG readout was acquired following the cessation of anti-seizure medication. Seizures and non-seizures were identified in 664 EEG files from the CHB-MIT database, which comprised 198 seizures from all patients. With 129 files involving one or more seizures, these data sets range in duration from one to four hours. A rate of 256 samples per second was used to record every EEG signal. In line with this, PhysioNet has the CHB-MIT dataset, which consists of 23 patients' multi-channel EEG signals. Additionally, it is a publicly accessible dataset with various EEG recordings that was obtained using an hour-long recording of the EEG signals using a conventional 10–20 system. At every recording, it is labelled with the beginning and ending of the seizure. Typically, a session of 23 electrodes is used to sample EEG signals at 256 Hz.

#### Bonn dataset

**figure 3:**
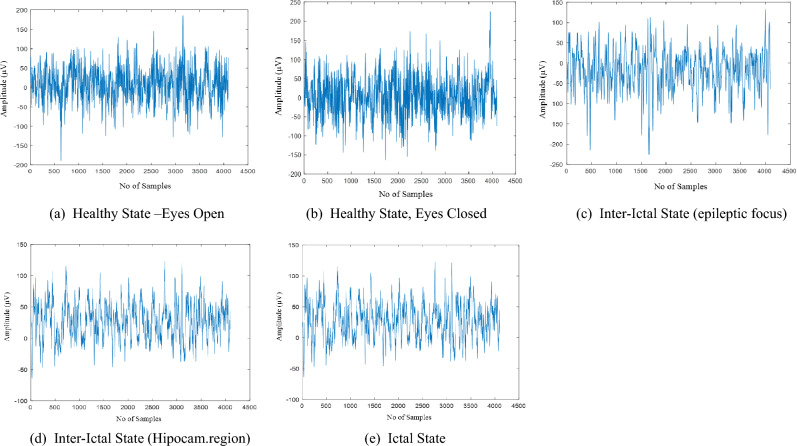
Sample EEG signals.

The Bonn University dataset description is given in Table 1. It is divided into five subsets, A, B, C, D, and E, each of which has about 100 single-channel segments. The data set utilised in this study was gathered by a University of Bonn research team and has been extensively used in studies on the detection of epilepsy with the sampling frequency of 173.61 Hz. The standard method of placing 10–20 electrodes was employed to record the EEG signals. There are 100 one-channel instances in each of the five sets (A to E) that make up the entire data collection. Five healthy participants were recorded with their eyes open (A) and closed (B) when they were relaxed and awake when the EEG data for Sets A and B were obtained. Sets C, D, and E comprised information from five patients. The EEG traces for set D were gathered in the epileptogenic zone. Set C was recorded in the other hemisphere of the brain, where the hippocampus is formed. Unlike Sets C and D, which contain EEG signals recorded during seizure-free periods (interictal) (ictal), Set E's EEG data were exclusively recorded during seizure activity. For both datasets, around 80% of data has been taken for training models and 20% of data is considered for testing the models. Then, the type of sample signals obtained from the dataset are shown in Fig. 3.

**Table 1:** Bonn dataset description.

Data	Set A	Set B	Set C	Set D	Set E
Type	Healthy	Healthy	Epileptic	Epileptic	Epileptic
State	Awake State with Eyes Open	Awake State with Eyes closed	Interictal	Interictal	Ictal
No of Channels	100	100	100	100	100
Electrode Placement	International 10–20 System	International 10–20 System	Hippocampus Opposite to Hemisphere	Within Epileptic Zone	Within Epileptic Zone

### Comparative analysis

For this evaluation, the University of Bonn dataset has been used to test the performance and efficiency of the filtering approach, feature extraction model, and optimization mechanism separately. Table 2 shows the Peak Signal to Noise Ratio (PSNR) and SD of the existing Blackman window and maximally flat filtering techniques. For the signals obtained from the Bonn dataset, the average SNR and SD values are estimated by implementing both existing and proposed approaches. Then, these results stated that the proposed FLHF approach provides the reduced average SNR and SD values by eliminating the noise based on the probability estimation using the rectangular format of mother wavelets. In the proposed work, two main issues such as noise removal and class imbalance are solved in the preprocessing stage by using the FLHF mechanism. It helps to remove the noise and improve SNR of the EEG signals. All the operations involved in the preprocessing stage helps to improve the SNR of EEG signals, and also it results in an improved classification performance.

**Table 2:** Average PSNR and SD of existing and proposed filtering approaches.

Filter	Average Peak SNR (dB)	SD
Blackman Window	5.2713	1.0530
Maximally Flat	6.0257	1.3298
Proposed FLHF	7.4462	1.3040

Table 3 shows the analysis of the proposed FD feature extraction model using the university of Bonn dataset, where the performance measures are evaluated for each FD feature. It is clearly described how the proposed system obtains the results without the features of Higuchi FD, Katz FD, instantaneous energy, teager energy, and Petrosian FD. For analysis, the feature extraction model without Higuchi FD offers the reduced performance values compared to the integration of all FD features. Hence, it is stated that the incorporation of all FD features could efficiently improves the performance of entire seizure detection system.

**Table 3:** Analysis of feature extraction.

Features	Accuracy	Sensitivity	Specificity	Precision	Recall	F-Measure	G-Mean
Feature extraction without Higuchi FD	94.4	97.87	92.30	88.46	97.87	0.9293	0.9505
Feature extraction without Katz FD	95	96.01	94.31	91.90	96.01	0.9392	0.9516
Feature extraction without Instantaneous energy	96.2	96.03	96.30	94.63	96.03	0.9533	0.9617
Feature extraction without teager energy	98.2	99.48	97.36	96.05	99.48	0.9774	0.9842
Feature extraction without Petrosian FD	93	95.36	91.50	87.67	95.36	0.9136	0.9341
Feature extraction with inclusion of all FD features	99.8	99.5	100	100	99.5	0.99	0.99

Depending on how the epileptic seizure manifests itself, there are various categories into which it can be divided. The ictal state refers to the period between the beginning of the seizure and its conclusion. After a seizure has ended, a postictal state begins and lasts for a short while. Interictal state, or regular brain activity, is distinguished from preictal state, or abnormal brain activity, which can begin 60 to 90 min before the onset of a seizure. Here, the average accuracy is estimated in Table 3 for these classes of the EEG signals. In this analysis, the accuracy is only evaluated for with and without feature extraction model, where the accuracy is separately calculated for the individual features as well as the inclusion of all FD features. According to the estimated results, it is observed that the accuracy can be greatly increased to 99.8% with the inclusion of all FD features. Similarly, the proposed epileptic seizure detection system is tested with and without optimization stages as shown in Table 4. Based on the analysis, it is observed that with the inclusion of optimization, the accuracy is increased to 98.9% in the proposed model. Moreover, these two analyses are performed for individually validating the effectiveness of feature extraction and optimization mechanisms.

**Table 4:** Parametric evaluation of the proposed system for with and without optimization techniques.

Measures	Without optimization	With optimization
Accuracy	97.5	98.9
Sensitivity	97.5	98.6
Specificity	97	99.4
Precision	95.6	99.2
Recall	97.5	98.6
F-Measure	0.966	0.983
G-Mean	0.973	0.985

Table 4 shows the parametric evaluation of proposed system with and without optimization techniques, where the analysis has been taken for the Bonn university dataset. Based on this analysis, it is stated that without optimization the performance values of all measures are decreased, when compared to the performance values of “with optimization”. Because, the GBSO technique finds both local and global optimum value based on the optimal position updation of grasshoppers. Here, the optimized value can be predicted based on the random jumping strategy, which helps to efficiently training the classifier for predicting the seizure with better performance outcomes.

### Overall performance analysis

**Figure 4:**
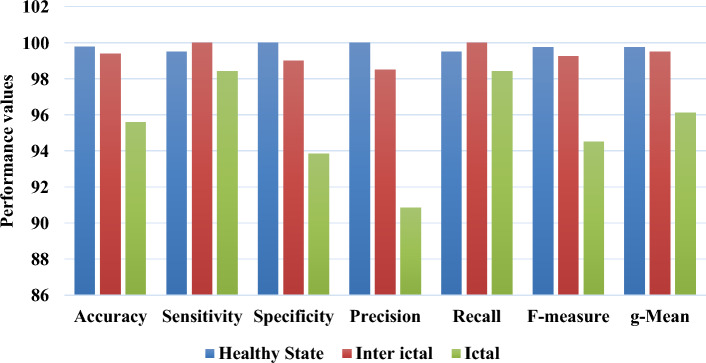
Performance analysis of proposed GBSO-TAENN technique.

Figure 4 presents the overall performance analysis of the proposed GBSO-TAENN mechanism, where the sensitivity, specificity, accuracy, precision, and F-measure measures have been computed. Typically, the sensitivity, specificity, and accuracy measures are used to test the classification efficiency of the detection system. Similarly, the parameters of precision, recall, g-mean and f-measure are also estimated in this work for ensuring the detection efficiency of the machine learning classifier. The sensitivity is defined based on the ratio of the number of TP rate and the value of TP with FN. Then, the specificity is defined by the ratio of the TN and TN with FP, and accuracy is estimated to determine that how the classifier could actually predict the seizure-affected signals based on the set of features. The measures are defined as follows:

37 $$Sensitivity = \frac{TP}{{TP + FN}}$$

38 $$Specificity = \frac{TN}{{TN + FP}}$$

39 $$Accuracy = \frac{TP + TN}{{TP + TN + FP + FN}}$$

40 $$Precision = \frac{TP}{{TP + FP}}$$

41 $$F - Measure = \frac{2TP}{{2TP + FP + FN}}$$

where TP indicates the True Positive, TN represents the True Negative, FP defines the False Positive, and FN represents the False Negative. Based on this analysis, it is observed that the proposed GBSO-TAENN model provides improved classification results with high accuracy, sensitivity, specificity, precision and f-measure values.

**Figure 5:**
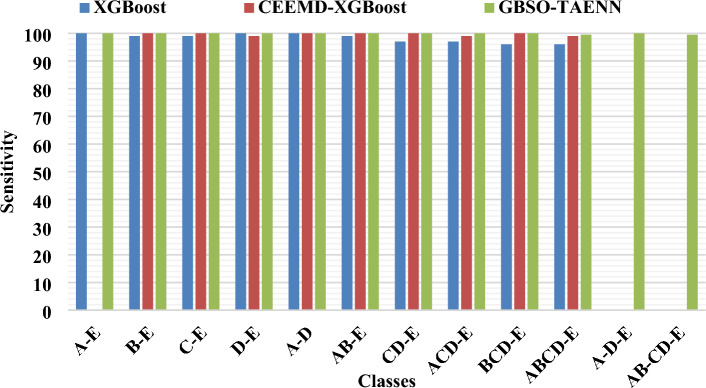
Comparative analysis of sensitivity between existing and proposed techniques.

**Figure 6:**
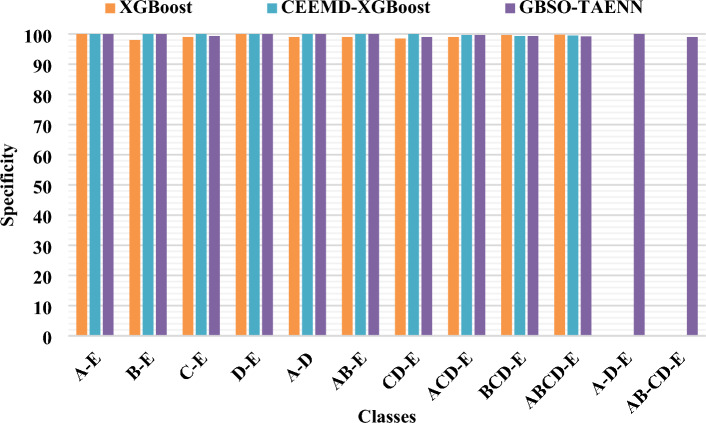
Comparative analysis of specificity between existing and proposed techniques.

**Figure 7:**
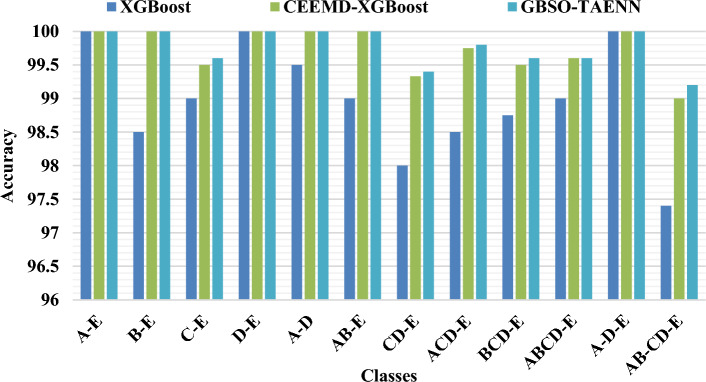
Comparative analysis of accuracy between existing and proposed techniques.

### Comparative analysis between existing and proposed techniques

Figures 5, 6 and 7 compares the existing XGBoost, CEEMD-XGBoost^33^ and proposed GBSO-TAENN mechanisms based on sensitivity, specificity, and accuracy measures. These parameters are computed for the different subsets of signals mentioned in the Bonn University dataset. In this analysis, around 12 number of classes have been considered for testing the results of both existing and proposed techniques. Also, the obtained values of both techniques are tabulated in Table 5 and Table 6. The evaluation shows that the proposed GBSO-TAENN technique outperforms the other techniques with increased performance values. Because it optimally selects the most relevant features by estimating fitness value with respect to the distance measure, which helps improve the classifier's overall performance.

**Table 5:** Analysis of sensitivity and specificity between existing and proposed techniques for different datasets.

Dataset	Cases	Classes	XG Boost		CEEMD-XGBoost		GBSO-TAENN	
			Sensitivity	Specificity	Sensitivity	Specificity	Sensitivity	Specificity
Bonn	I	A-E	100	100	100	100	100	100
	II	B-E	99	98	100	100	100	100
	III	C-E	99	99	99	100	100	99.33
	IV	D-E	100	100	100	100	100	100
	V	A-D	100	99	100	100	100	100
	VI	AB-E	99	99	100	100	100	100
	VII	CD-E	97	98.5	99	100	100	99
	VIII	ACD-E	97	99	100	99.67	100	99.67
	IX	BCD-E	96	99.67	99	99.33	100	99.33
	X	ABCD-E	96	99.75	99	99.50	99.5	99.2
	XI	A-D-E	–	–	–	–	100	100
	XII	AB-CD-E	–	–	–	–	99.5	98.99
CHB-MIT	XIII	Normal/Seizure	93.46	92.83	95.70	95.89	99	98.9

**Table 6:** Accuracy analysis of existing and proposed techniques for two datasets.

Dataset	Cases	Classes	XG Boost	CEEMD-XGBoost	GBSO-TAENN
			Accuracy	Accuracy	Accuracy
Bonn	I	A-E	100	100	100
	II	B-E	98.50	100	100
	III	C-E	99	99.50	99.6
	IV	D-E	100	100	100
	V	A-D	99.50	100	100
	VI	AB-E	99	100	100
	VII	CD-E	98	99.33	99.4
	VIII	ACD-E	98.50	99.75	99.8
	IX	BCD-E	98.75	99.50	99.6
	X	ABCD-E	99	99.60	99.6
	XI	A-D-E	100	100	100
	XII	AB-CD-E	97.40	99	99.2
CHB-MIT	XIII	Normal/Seizure	93.14	95.79	99.1

**Figure 8:**
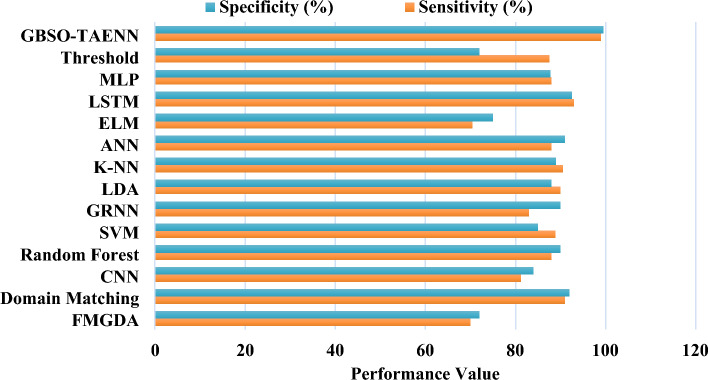
Overall comparative analysis between existing and proposed classification techniques.

In Fig. 8, some of the recent state-of-the-art model approaches including both machine learning and deep learning are compared with the GBSO-TAENN algorithm for proving the superiority of the proposed model. For this analysis, the parameters such as sensitivity (%), specificity (%), and average anticipation time (m) are taken into consideration. Since, the accuracy of classifier is highly relies on the parameters of sensitivity, specificity, and time. Therefore, the suggested existing and proposed GBSO-TAENN models are validated and compared in this table. The findings indicated that the proposed algorithm overwhelms the conventional approaches with increased sensitivity, specificity, and reduced time due to the proper operations such as filtering, feature extraction, optimization and classification.

Figure 8 compares the sensitivity and specificity values of various machine learning-based classification approaches used for epileptic seizure detection. The techniques taken for this analysis are thresholding, MLP, LSTM, ELM, ANN, KNN, LDA, GRNN, SVM, RF, CNN, domain matching, and FMGDA^35–37,45^. The evaluation proves that the proposed GBSO-TAENN technique provides an increased performance value by accurately classifying the seizure-affected signals from the given dataset.

**Figure 9:**
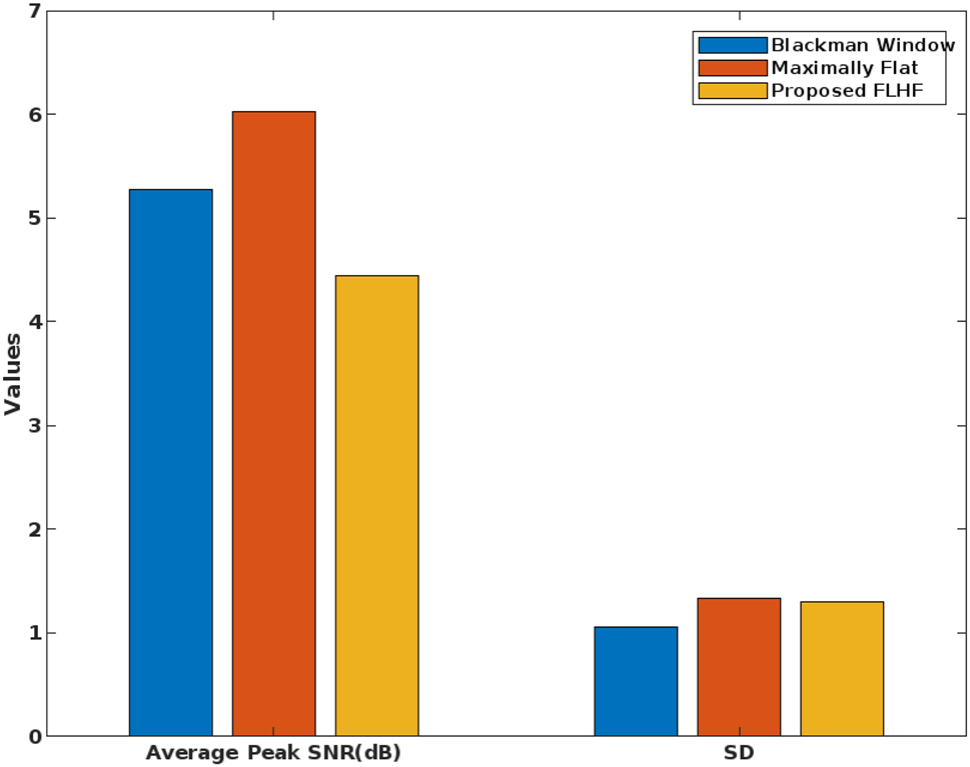
Performance of the filtering technique.

**Figure 10:**
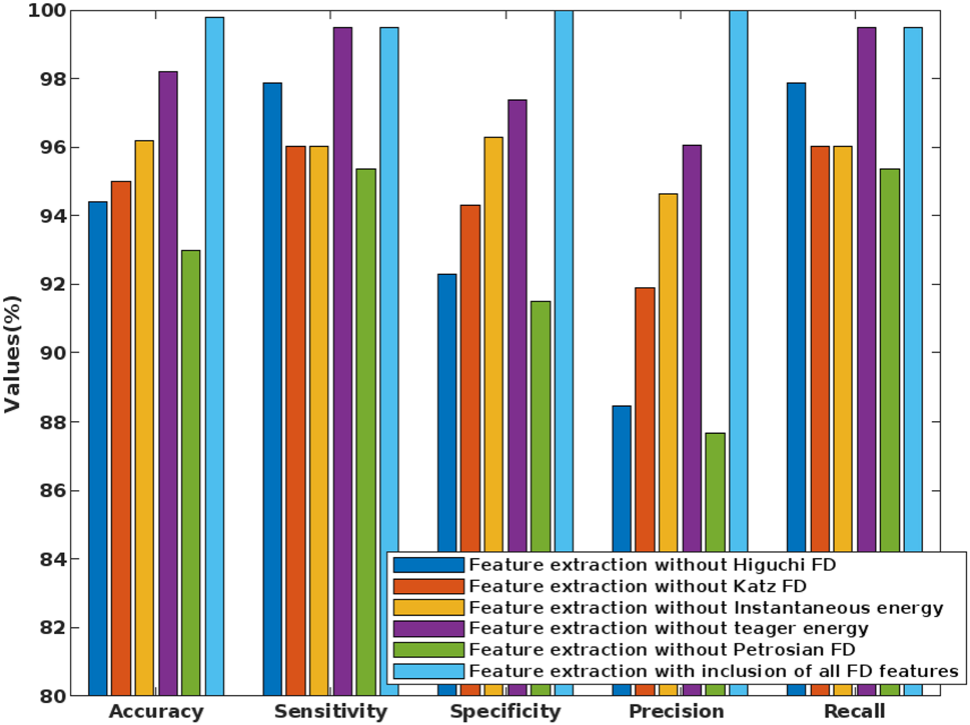
Performance analysis with and without feature extraction.

**Figure 11:**
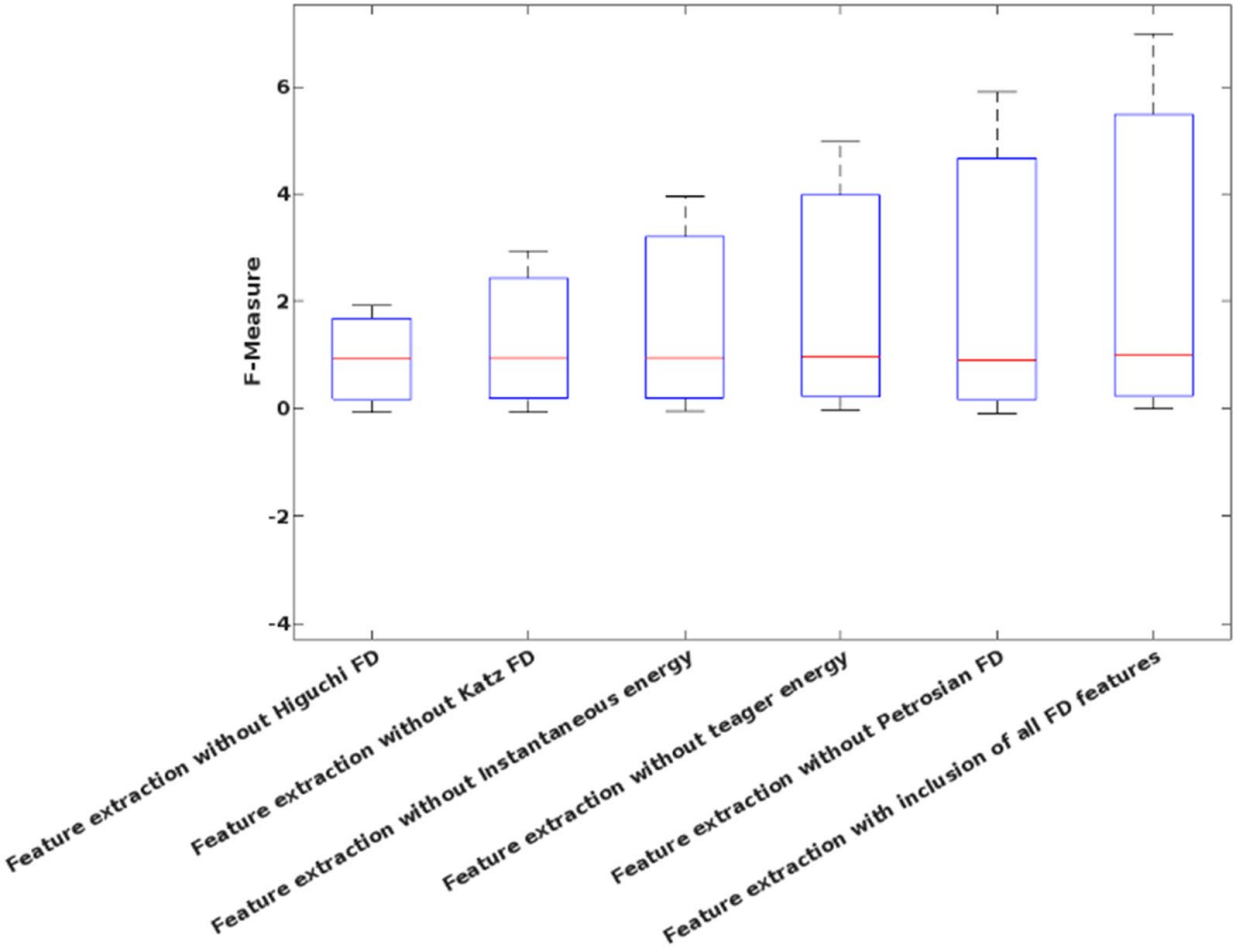
F-measure for with and without feature extraction.

**Figure 12:**
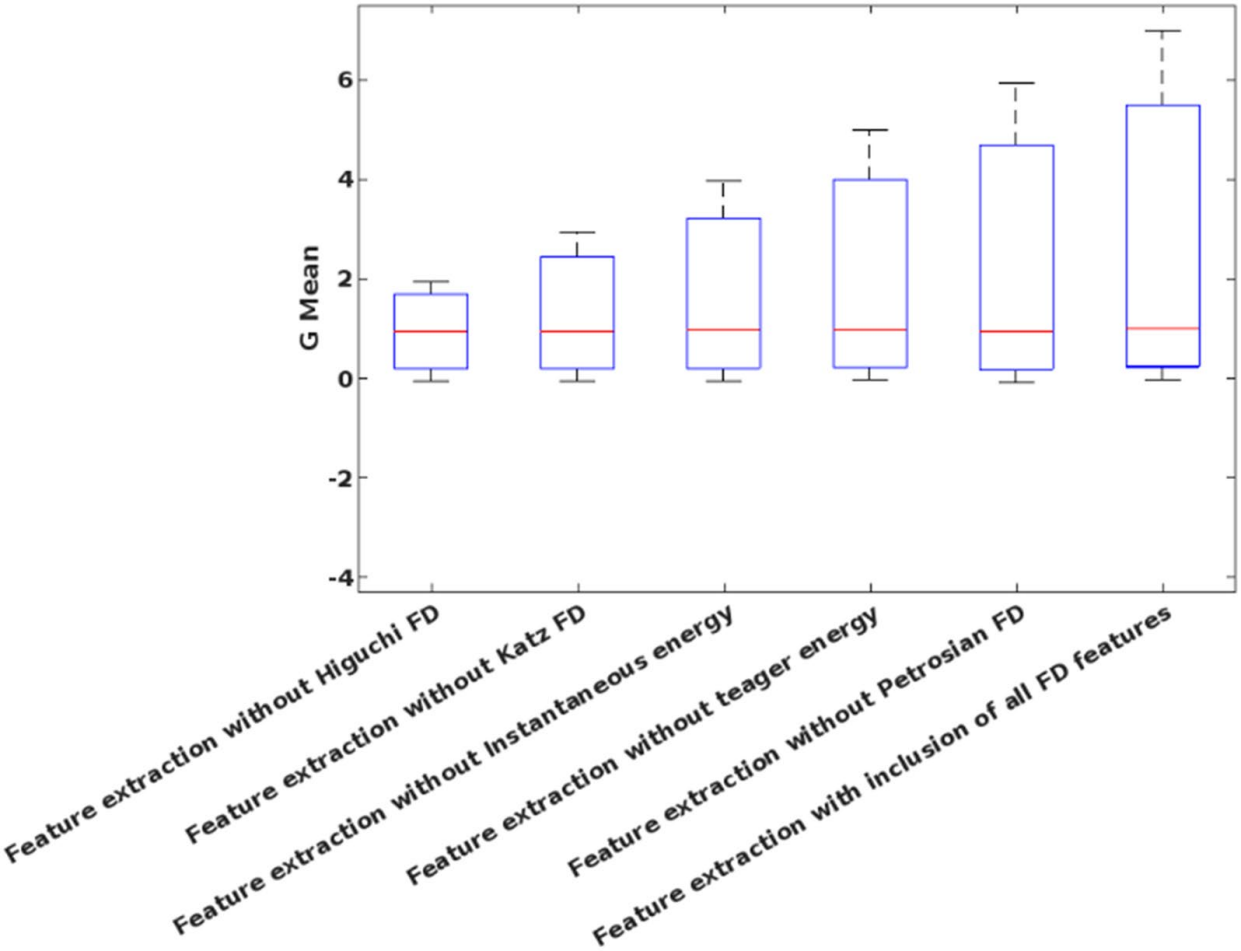
G-Mean for with and without feature extraction.

**Figure 13:**
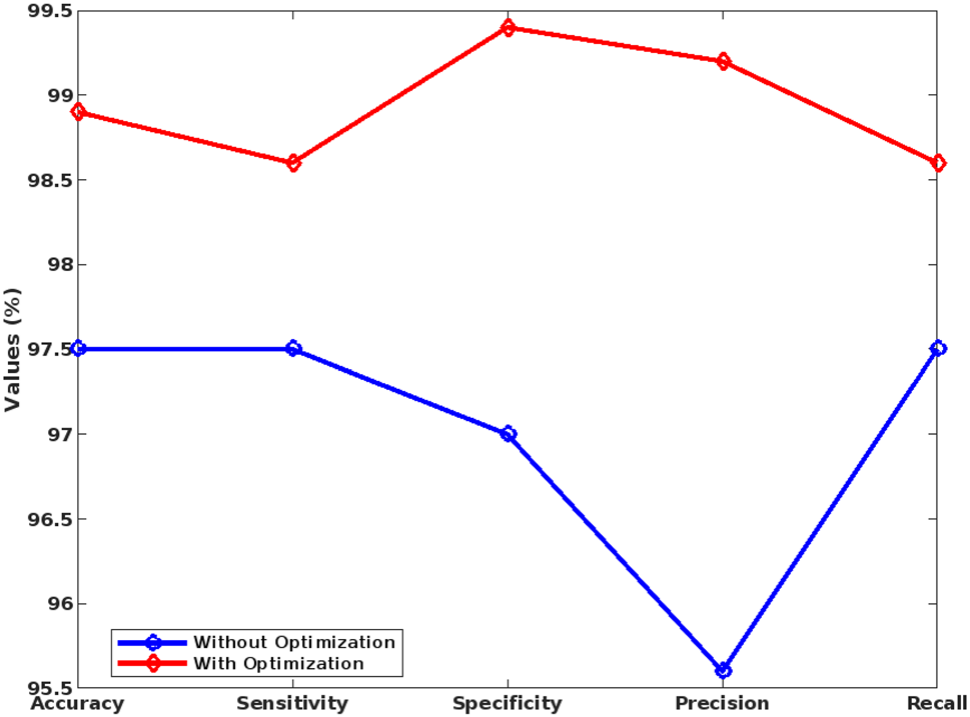
Performance analysis with and without optimization.

**Figure 14:**
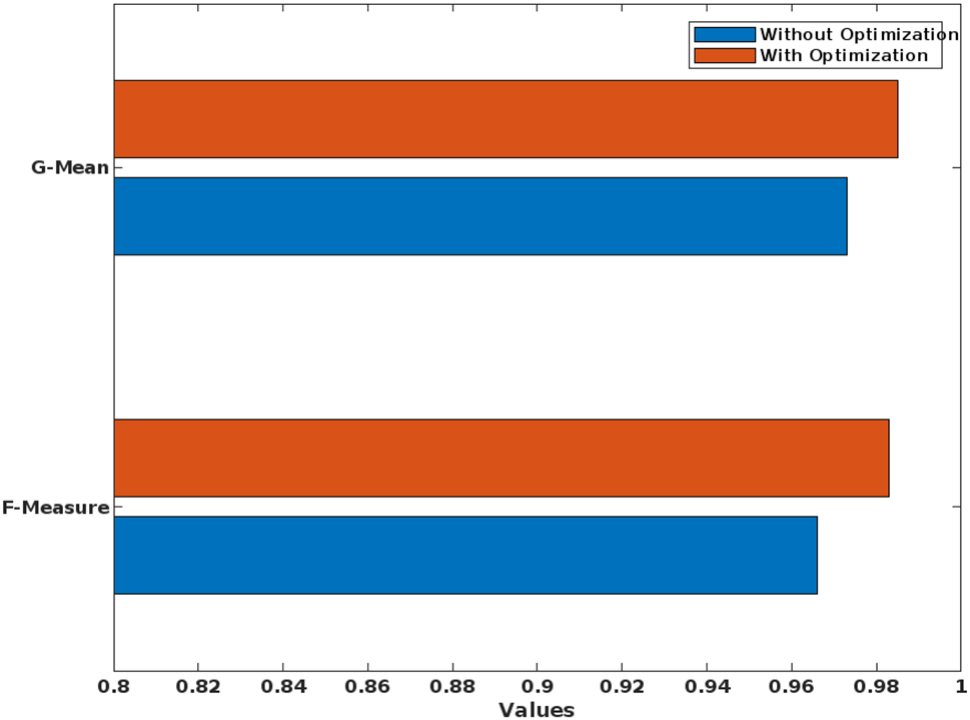
F-measure and g-mean values for with and without optimization.

Figure 9 validates the performance of the conventional and proposed filtering technique based on the parameters of average peak SNR, and standard deviation, which includes existing filtering techniques of blackman window and maximally flat. According to this analysis, it is identified that the proposed FLHF technique outperforms the other approaches with better SNR, and SD values. Figure 10 compares the performance of the proposed methodology with and without feature extraction. The obtained results depict that the proposed detection system provides an improved performance values with the inclusion of FD feature extraction technique. Consequently, Figs. 11 and 12 compares the F-measure and G-mean values of the proposed methodology with and without feature extraction techniques. The obtained results state that the proposed model provides an improved F-measure and G-mean values, when it is incorporated with all features. According to this evaluation, it is observed that the feature extraction method plays a vital role in the proposed system, since it supports to obtain an improved classification performance. Similarly, the performance analysis of the proposed mechanism is validated with and without optimization techniques as shown in Figs. 13 and 14. Based on the results, it is analyzed that the proposed technique provides an improved results with the inclusion of optimization.

**Figure 15:**
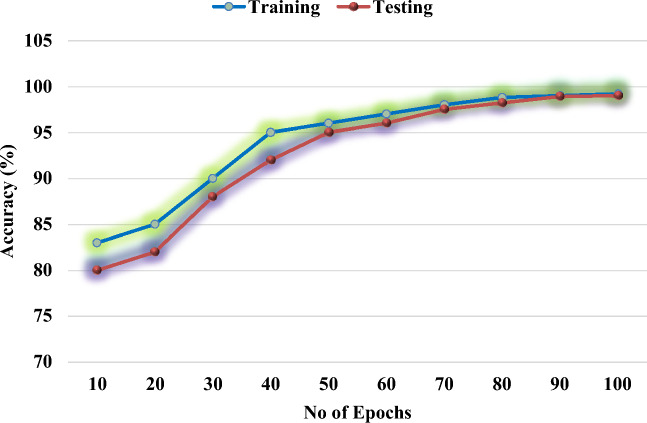
Training and testing accuracy.

Figure 15 shows the training and testing performance of the proposed GBSO-TAENN classification technique with respect to varying number of epochs. According to the observed results, it is determined that the proposed model provides an improved training and testing outcomes with the inclusion of filtering, FD feature analysis and GBSO techniques.

## Conclusion

In this work, a novel classification method called GBSO-TAENN for identifying epileptic seizures based on provided EEG signals is presented. This paper's primary contribution is the creation, via sophisticated signal processing techniques, of an intelligent and effective epileptic seizure detection system. Feature extraction, feature selection, and classification algorithms are used in the development of multiple EEG signal processing frameworks in conventional works. However, most methods have issues with large computing overhead, poor accuracy, and lengthy forecast times for seizures. Thus, the goal of the proposed work is to create a framework that is both straightforward and computationally effective for identifying and categorizing epileptic seizures based on EEG signals. Even though epileptic seizures are uncommon, they are extremely important to diagnose and treat because of the influence they have on social interaction, physical communication, and patient sentiment. To improve the signal quality, the EEG signals are first preprocessed. Once the data has been preprocessed, an optimal subset of features is derived using the FD. The GBSO is then utilised to further optimise the FD and decrease classification time while improving accuracy. In order to predict abnormalities in the provided EEG signal, the classification is ultimately carried out using the TAENN algorithm. The unique contribution of this work is the accurate disease prediction it achieves by using sophisticated and effective signal processing techniques such as preprocessing, feature extraction, optimization, and classification. The unique inertia weight updation is carried out during feature selection, which enhances optimisation performance and convergence. Subsequently, the Deep Neural Network (DNN), back propagation method, and regular spatiotemporal neural network are used to implement the TAENN. Therefore, an automated system for detecting epileptic seizures would be more suited to use GBSO-TAENN in conjunction. This work's primary goal is to correctly categorise the input EEG signal as either normal or seizure-related. Several soft computing approaches have been used in this work to achieve this goal, and the FLHF approach is used for signal decomposition and normalisation. The attributes of Higuchi, Katz, Sevcik's, instantaneous energy, petrosian FD, and teager energy are then extracted using the FD analysis. The efficacy of the seizure prediction and classification system is enhanced by these feature vectors. Next, in order to further reduce the dimensionality of feature vectors, the fitness value is utilised in conjunction with the GBSO process to select the best features. Ultimately, the TAENN-based classifier is used to identify whether the provided EEG signal is seizure-affected or normal. Here, the effectiveness of this detection method has been evaluated using two distinct benchmark datasets. Additionally, in order to demonstrate the improvement of the suggested method, the acquired findings are contrasted with a few other traditional processes. According to the evaluation, the GBSO-TAENN approach raised the accuracy to 99%, specificity to 99.5%, and sensitivity to 99%. These findings then demonstrated that, for both datasets, the GBSO-TAENN methodology performs better than the other methods. By using cutting-edge deep learning models for the medical diagnosis systems, this study can be expanded in the future.

## Data Availability

The datasets used and/or analysed during the current study available from the corresponding author on reasonable request.
